# gscramble: Simulation of Admixed Individuals Without Reuse of Genetic Material

**DOI:** 10.1111/1755-0998.14069

**Published:** 2025-01-12

**Authors:** Eric C. Anderson, Rachael M. Giglio, Matthew G. DeSaix, Timothy J. Smyser

**Affiliations:** ^1^ Fisheries Ecology Division Southwest Fisheries Science Center, National Marine Fisheries Service Santa Cruz California USA; ^2^ Department of Fish, Wildlife, and Conservation Biology Colorado State University Fort Collins Colorado USA; ^3^ Department of Biology Colorado State University Fort Collins Colorado USA; ^4^ United States Department of Agriculture, Wildlife Services National Wildlife Research Center Fort Collins Colorado USA

**Keywords:** genetic stock identification, mixture deconvolution, permutation testing, power analysis, R package

## Abstract

While a best practice for evaluating the behaviour of genetic clustering algorithms on empirical data is to conduct parallel analyses on simulated data, these types of simulation techniques often involve sampling genetic data with replacement. In this paper we demonstrate that sampling with replacement, especially with large marker sets, inflates the perceived statistical power to correctly assign individuals (or the alleles that they carry) back to source populations—a phenomenon we refer to as resampling‐induced, spurious power inflation (RISPI). To address this issue, we present gscramble, a simulation approach in R for creating biologically informed individual genotypes from empirical data that: (1) samples alleles from populations *without* replacement and (2) segregates alleles based on species‐specific recombination rates. This framework makes it possible to simulate admixed individuals in a way that respects the physical linkage between markers on the same chromosome and which does not suffer from RISPI. This is achieved in gscramble by allowing users to specify pedigrees of varying complexity in order to simulate admixed genotypes, segregating and tracking haplotype blocks from different source populations through those pedigrees, and then sampling—using a variety of permutation schemes—alleles from empirical data into those haplotype blocks. We demonstrate the functionality of gscramble with both simulated and empirical data sets and highlight additional uses of the package that users may find valuable.

## 1 Introduction

Genetic clustering algorithms are commonly used to describe genetic structure among populations, reflecting the cumulative effects of isolation, connectivity (i.e., genetic migration), selection, and genetic drift. The ability of such algorithms to differentiate populations is limited by the extent of genetic differentiation among populations and the capacity to resolve those differences given the available marker set and sample sizes from the respective populations. Based on an initial description of genetic structure, ecologists are frequently interested in describing patterns of genetic movement among populations, often inferred from the mismatch between the sampling location and genetic origins of a given individual (Paetkau et al. 1995; Wilson and Rannala 2003). Genetic movement has many implications, such as influencing evolutionary processes, maintaining genetic variation through gene flow, driving disease dynamics and affecting patterns of invasive species expansion (Huestis et al. 2019; Estoup and Guillemaud 2010).

In using clustering algorithms to describe genetic structure among natural populations, researchers frequently identify individuals of mixed ancestry—individuals with proportions of their genome attributed to multiple subpopulations, sensu Pritchard, Stephens, and Donnelly (2000). Various processes could contribute to the appearance of mixed ancestry in a clustering analysis. Most directly, individuals may represent the influences of gene flow among populations, occurring in recent or prior generations, with the observed complex ancestry patterns accurately representing contributions from multiple sampled populations. However, similar patterns may result from functionally different patterns of population structuring. For example, such patterns might be expected when clinal systems governed by isolation by distance are described as discrete genetic clusters. Alternatively, individuals of mixed ancestry could reflect immigration from outlying populations (e.g., exogenous immigration), with true source populations not sufficiently represented in the sample to be among the characterised K subpopulations. Furthermore, the characterisation of admixed individuals may reflect a limitation of the statistical power of the genetic data (as a function of both the marker set and sample size) to resolve the true underlying patterns of genetic structure. The challenge for researchers then becomes correctly identifying the ecological and/or statistical processes by which observed complex ancestry patterns were created.

A best practice for evaluating the behaviour of genetic clustering algorithms on empirical data is to conduct parallel analyses on simulated data that mirror the empirical data set as closely as possible (Vähä and Primmer 2006; Anderson, Waples, and Kalinowski 2008; Latch et al. 2011) while providing the benefit of known parameters (e.g., $F_{\mathrm{ST}}$, admixture fractions, etc.) that can be compared to estimates. Techniques to simulate individual genotypes in this context frequently use sampling with replacement from the observed allele frequencies (see, for example, Nielsen, Bach, and Kotlicki 2006; Kinziger et al. 2008). In this approach, allele frequencies are estimated for each population (or cluster) from the samples, and then new genotypes are simulated at each locus by drawing, for each simulated diploid individual, two gene copies from a multinomial distribution with two trials and cell probabilities given by the estimated allele frequencies. Such a simulation is akin to assuming that the population is of infinite size with allele frequencies given by those observed in the sample; however the allele frequencies in the sample differ from those in the actual population because of the variation from random sampling. As a consequence, this sort of sampling with replacement implicitly increases the pairwise genetic distance among simulated samples, relative to the observed samples, and inflates the perceived statistical power to correctly assign individuals (or segments of the genome) back to source populations. In effect, simulations that sample with replacement from the samples are assessing how well one could assign individuals to populations that have the same allele frequencies as the samples, rather than the same allele frequencies as the populations *from which the samples were drawn*, which is what is needed. We refer to this phenomenon as resampling‐induced, spurious power inflation (RISPI). Anderson, Waples, and Kalinowski (2008) used simulations to document this type of artefact in the context of genetic stock identification (GSI), a type of clustering application in which individuals are constrained to have all their ancestry from a single subpopulation.

Beyond simply evaluating the resolution of discrete populations in a simulated framework, researchers may also be interested in how individuals of admixed ancestry are genetically characterised given the statistical power of the available dataset and the analysis methods used. Recognising that connectivity among genetically distinct populations may be relatively rare, additional insight into the frequency of immigration can be gained by integrating across generations to identify both direct migrants and descendants of migrants that is, F1 hybrids or advanced hybrids reflecting the effect of backcrossing across multiple generations [*BC*_1_,…,*BC*_*x*_]. Evaluating the results and resolution of genetic clustering algorithms in the context of data that are expected to reflect a history of hybridisation requires simulation of explicit hybrids. Such simulations are the purpose of the software hybridlab (Nielsen, Bach, and Kotlicki 2006).

hybridlab was conceived at a time when the data sets used in molecular ecology universally included very few markers, such that it was reasonable to assume the markers were unlinked, and, hence, independently segregating. Today, however, with data sets of thousands of markers, that assumption is badly violated—alleles on the same chromosome are likely to be segregated together into the next generation. Simulating linked loci as independent creates simulated hybrids, beyond the F1 category, with artificially low variance in admixture fraction, leading to the false interpretation that different hybrid categories are more easily distinguished than they are. Therefore, in evaluating descendants of migrants within a simulated framework, it is imperative to take into consideration the pattern of linkage among loci available for analysis. This was done recently to assess the power of a SNP panel for identifying descendants of escaped, farm‐raised Atlantic salmon (Wringe et al. 2019); however the method used (described at https://eriqande.github.io/SalarHybPower/) was developed in an *ad hoc* fashion, was not easily extended to complex hybridisation scenarios, and is not user‐friendly.

In response to these needs, we developed gscramble—a simulation approach that samples alleles from respective populations without replacement, thus maintaining genetic differences between the clusters commensurate to those in the empirical dataset, and eliminating the effect of RISPI. Further, by simulating individual genotypes based on user‐defined pedigrees of arbitrary complexity, gscramble allows for the simulation of admixed genotypes resulting from any conceivable category of (non‐inbred) hybrid, while allowing the tracking of haplotype blocks from source populations within the specified pedigree. By integrating species‐specific recombination rates, gscramble simulates biologically informed genotypes that mirror empirical data. We develop and illustrate gscramble through the use of single nucleotide polymorphism (SNP) genotypes, though it is equally applicable to data sets with hundreds or thousands of microhaplotypes (Baetscher et al. 2018) or microsatellites (Zhan et al. 2017).

## 2 Methods

### 2.1 Two Introductory Motivating Examples

Before we proceed to a description of the methodology used by gscramble, we demonstrate two important points through two simple simulations. In the first, we show that using a standard sampling‐with‐replacement approach induces RISPI when estimating admixture fractions. In the second, we show the effect of physical linkage on the distribution of admixture fractions of hybrid individuals, which vividly shows why it is not acceptable, with large numbers of markers, to simulate the genotypes of hybrid individuals assuming no physical linkage.

#### 2.1.1 RISPI When Estimating Admixture Fractions

To construct this simple illustration of sampling‐with‐replacement induced RISPI, we used the R programming language (R Core Team 2023) to simulate allele frequencies for L biallelic loci in a single population from a $\text{Beta}\left(1,8\right)$ distribution and then simulated two samples, A and B, each of size N diploid genotypes. The genotypes in A and B were simulated from the same allele frequencies assuming Hardy–Weinberg equilibrium.

In this case, each set of N individuals is a sample from exactly the same population, so there is clearly no basis for performing population assignment between A and B; however, we treated samples A and B as if they were sampled from two potentially different populations, and simulated 9n new individuals: n new individuals at each of the nine values of $q_{A}$, the admixture fraction for population A, in $\left\{0,\frac{1}{8},\frac{1}{4},\frac{3}{8},\frac{1}{2},\frac{5}{8},\frac{3}{4},\frac{7}{8},1\right\}$. We simulated these genotypes according to the ‘admixture model’ used in *structure* (Pritchard, Stephens, and Donnelly 2000). Briefly, at each locus, independently, the origin of each gene copy was simulated to be from population A with probability $q_{A}$ and from population B with probability $1-q_{A}$. Subsequently, the allelic types of gene copies from A (B), at a locus, were simulated by sampling with replacement from the alleles at that locus in sample A (B). We then analysed all the genotypes (9n+N+N) using ADMIXTURE with K=2. On each data set we performed a supervised analysis in which the original N samples from each population were provided as learning samples, and also an unsupervised analysis when the origin of the samples from A and B were regarded as unknown.

For each combination of $L\in \left\{10^{2},10^{3},10^{4},10^{5}\right\}$, $N\in \left\{25,50,100,250\right\}$ and $n\in \left\{3,12,24\right\}$ we conducted multiple replicates of the entire process of simulating samples A and B, simulating the 9n additional individuals, and then analysing them with ADMIXTURE. We conducted R replicates so that, for each combination of L, N, and n we had simulated Rn=480 new individuals at each $q_{A}$ value; thus, R=160 replicates when n=3, R=40 when n=12, and R=20 when n=24.

Because the simulations were done in a naïve way that effectively assumes that the population allele frequencies are identical to those observed in the samples themselves, we hypothesized that ADMIXTURE would return estimates of $q_{A}$ that were centred around the simulated values, even though samples A and B were drawn from the same population. In other words, we expected to observe RISPI. We further expected that ADMIXTURE would return estimates of $q_{A}$ that were closer to the simulated values when N was smaller and the number of loci was larger—attributes that magnify the effect of RISPI. We summarised the results with boxplots of the ADMIXTURE‐inferred $q_{A}$ values, for the 480 newly simulated individuals (and for the original samples, A and B, in the case of unsupervised clustering), for each combination of L, N, and n.

#### 2.1.2 Physical Linkage in Hybrid Individuals

We first simulated the genotypes of hybrids between two species, a and b, considering $F_{2}$'s and first‐ through fourth‐generation backcrosses in each direction, erroneously treating each locus as if it were independently segregating (as done, e.g., in hybridlab). Thus, each locus independently is simulated to have a genotype of 2, 1 or 0 gene copies originating from species a [e.g., genotypes of $\left(a,a\right)$, $\left(a,b\right)$, or $\left(b,b\right)$] according to the expected probability of each genotype given the hybrid category (see Anderson and Thompson 2002). 48,000 hybrids were simulated at each number of loci, L, in $\left\{10^{2},10^{3},10^{4},10^{5}\right\}$. The admixture fraction of each simulated individual was recorded as the fraction of all 2L gene copies originating from species a.

We also simulated the genomes of 48,000 individuals of each hybrid category using gscramble's segregate() function, as described below, taking as an example, a species with 18 chromosomes across 2.35 gigabases of total physical genome length and sex‐averaged recombination rates the same as for pigs, as estimated by from Tortereau et al. (2012). (We chose a pig‐like genome here, as wild pigs form one of our example empirical data sets for illustration, below.) In this case, we recorded the admixture fraction of each simulated individual as the cumulative length of all genome segments that originated from species a, divided by the total genome length. This provides the distribution of the true admixture fraction when physical linkage of markers is not ignored.

All the admixture fraction distributions were summarised by plotting them as histograms so they could be compared easily.

### 2.2 Genomic Simulation Pedigrees

Our approach for simulating admixed individuals without creating RISPI is motivated by the traditional cross‐validation approach for assessing the power of mixed stock analysis (e.g., the ‘Monte Carlo cross‐validation’ approach described in Moran and Anderson 2019, 555). In the cross‐validation approach, individuals are removed from the reference samples and placed in a mixture sample of ‘unknowns’, whose origin is inferred using the remaining individuals in the reference samples. A key feature of this approach is that no new genetic material is being created by sampling with replacement; rather the genetic material of all individuals in the original sample is being used—either within the remaining individuals in the reference samples or within the individuals set aside as a test set in the simulated mixture.

Our scheme, described here, extends the traditional cross‐validation approach by simulating the descent of chromosomal segments through a user‐specified pedigree to create admixed individuals. By imposing certain constraints on the pedigree, our framework ensures that all the genetic material within the original individuals is used to either create admixed individuals or is retained within reference individuals (i.e., those that are purely of one putative subopulation), and yet no new genetic material is created by sampling with replacement from the original samples.

We refer to the structure used to implement this cross‐validation simulation of admixed individuals as a *genomic simulation pedigree* or GSP. Like any pedigree, a GSP includes a set of founders (individuals that have no parents specified in the pedigree), and it includes non‐founders; however, we introduce an additional node type in the GSP that represents samples taken from the non‐founders. These samples are the simulated admixed individuals. A GSP must have no inbreeding loops, since any inbreeding loop indicates that more than one copy of a gene in an ancestor may be created among its descendants, which is a type of sampling with replacement. Furthermore, in a GSP, the amount of genetic material taken from the samples at the sample nodes should be equal to the amount of material in the founders, to ensure that all available data are being used in the simulations to predict the power for estimating admixture fractions.

**FIGURE 1:**
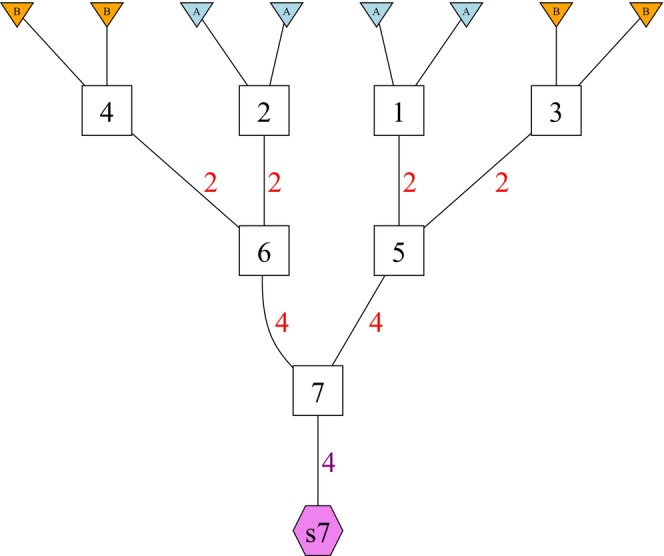
A genomic simulation pedigree (GSP) for simulating *F*2 individuals. See text for detailed explanation.

Figure 1 shows an example GSP for the simple case of simulating $F_{2}$ individuals.

In this figure, squares denote founder and non‐founder nodes in the pedigree. There are four founders in the pedigree, individuals 1–4. The triangles above each of those founders denotes, in colour and text, the population of origin of each of the two genomes within each founder. Thus, individuals 1 and 2 are founders purely from population A, and 3 and 4 are purely from population B. The pink hexagon labelled s7 is the sample node. It represents samples that are taken from non‐founder node 7, which, from the structure of the pedigree, clearly represents an $F_{2}$ hybrid between populations A and B. The red numbers on edges above and leading into each non‐founder node indicate the number of gametes that get segregated from the parent node (at the top of the edge) to the daughter node (at the bottom of the edge). This emphasises that, in dropping genes through a GSP, in contrast to a traditional pedigree, each parent node may segregate multiple gametes to a daughter node. See the text for further explanation. Each non‐founder node x will have exactly two edges coming into it from above (from its two parents), and we denote the numbers associated with those edges as $G_{x,1}^{+}$ and $G_{x,2}^{+}$. For example, node 6 has $G_{6,1}^{+}=G_{6,2}^{+}=2$. A founder individual may have, at most, 2 edges coming downward out of it, while a non‐founder individual may have more or less than two edges coming downward out of it. Letting $e_{x}$ be the number of downward edges out of node x to its $e_{x}$ non‐founder daughter nodes, we denote the number of gametes segregated through each edge as $G_{x,1}^{-},\ldots ,G_{x,e_{x}}^{-}$. For example, at non‐founder node 5, $e_{5}=1$ and $G_{5,1}^{-}=4$. Finally, the purple number adjacent to the edge leading into the sample node indicates how many sample individuals are drawn from individual node 7. Any non‐founder individual node can have a maximum of one edge leading to a sample node. We denote the number of samples emanating from node x as $S_{x}$. In our example $S_{7}=4$ indicates that four $F_{2}$ individuals are created from a single simulation on this GSP. It is important to understand that, apart from the founder nodes, the individual nodes in a GSP do not necessarily represent only a single individual. Specifically, as we are preserving all genetic material, multiple gametes may get segregated through each non‐founder node in a GSP.

Genetic material is segregated through a GSP using these steps, done upon each node in an order such that the steps are run on each node's parents before being run on the node itself:

1. Each genome in a triangle above a founder node represents a gamete carrying a single, haploid copy of the genome. These gametes are united within the founder individual and then recombination occurs between each chromosome with probability $\frac{1}{2}$ and within each chromosome according to a user‐specified recombination map to create two new gametes, which are recombined versions of the original ones. These two gametes are then segregated at random down the edge (or edges) from the founder node. Upon each edge, i from founder node f, a number, $G_{f,i}^{-}$, of gametes are segregated.
2. Each non‐founder node, x, will have exactly two edges coming into it—one from each parent. Each of the $G_{x,1}^{+}$ gametes coming in from the first edge is united with a single one of the $G_{x,2}^{+}$ gametes coming in on the second edge (in a random order).
3. If a non‐founder node x has an edge to a sample node, a randomly chosen $S_{x}$ of the united pairs of gametes are delivered to the sample node to constitute the $S_{x}$ samples taken from individual node x. Any remaining united pairs of gametes are treated as in (4) below.
4. United pairs of gametes (including those remaining after delivery to a sample node) in each non‐founder node x are segregated into gametes with recombination, and each of these gametes is assigned, in random order, to the gamete edges proceeding downward out of the node, with the number of gametes assigned to the $i^{\mathrm{th}}$ downwards edge being $G_{x,i}^{-}$.

At the end of this process, the samples simulated (as united pairs of gametes) into the sample nodes will contain all of the genetic material (and no more) found among the founders, but that material will have been segregated and recombined according to the pedigree. Thus the samples obtained from a GSP can be simulated with the expected admixture fractions for any hybrid category that can be specified by a pedigree. They have also been simulated in a way that (1) reflects physical linkage—each individual inherits segments of the genome simulated using a recombination map; (2) does not duplicate any genetic material—the sampling of genetic material is entirely without replacement so it will not induce RISPI and (3) no genetic material has been lost—all the genetic material of the founders is represented in the samples, maximising the number of simulated individuals and markers available to use in downstream analyses.

**FIGURE 2:**
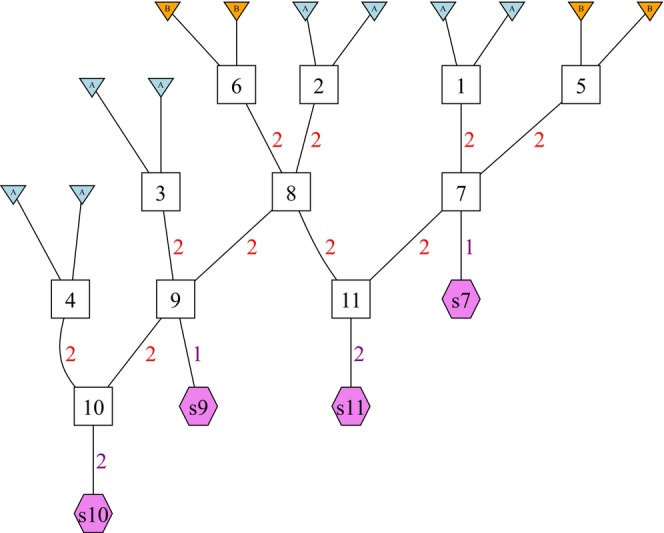
An example GSP generated by create GSP() that produces four different admixture categories: One *F*1 in sample node s7; two *F*2's in sample node s11; one *BC*1 in sample node s9; and two *BC*2's in sample node s10.

In the gscramble package there is a convenience function called create_GSP() that will create GSPs for samples up to and including second‐generation backcrosses (an example appears in Figure 2); however, gscramble is designed to handle user‐specified GSPs of arbitrary complexity. When creating a unique GSP, there are several requirements to ensure that the GSP is valid and the simulation can execute successfully. Writing F for the set of all founder nodes, N for the set of all non‐founder nodes, and $N_{s}$ for all non‐founder nodes that are not parents of other non‐founders, but are adjacent to a sample node, $N_{e}$ for all non‐founder nodes that have non‐founder node daughters, but no sample nodes, and $N_{s,e}$ for all non‐founder nodes that are parents of non‐founder nodes and also adjacent to sample‐nodes, the necessary conditions for a valid GSP, in addition to having no inbreeding loops, are as follows:
(1)

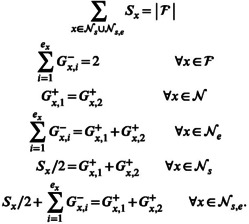




In words, the separate lines above specify that (1) the number of samples must equal the number of founders; (2) exactly two gametes must be segregated from each founder; (3) the number of gametes to a non‐founder along the edges from its parents must be equal; (4) the number of gametes going downward, out of a non‐founder with no sample daughters, must equal the number of gametes coming into that non‐founder; (5) the total number of gametes entering a non‐founder with only sample daughters must be half the number of sample daughters and (6) the number of gametes entering non‐founders with both sample daughters and non‐founder daughters must equal half the number of its sample daughters plus the sum of the number of gametes segregated along each edge to all of its non‐founder daughters.

Before simulating from a GSP, gscramble checks to ensure the GSP satisfies these validity conditions. The simulation of genomic segments from founders to samples in a GSP is performed by the gscramble function segregate(), which requires as input a list of GSPs through which to segregate genomic segments, and, for each such GSP, a table that maps the population labels on each GSP (in the inverted triangles atop the founders) to the different sampled groups of individuals.

### 2.3 Permutation Procedures

gscramble uses a GSP to segregate segments of genetic material from the founders to the samples. At the end of segment simulation, the genome of each sample is recorded as a mosaic of segments that derive from different founders. In order to create a data set of markers in each sample, it is necessary only to identify the alleles that occurred on each segment within the founders and propagate those alleles to the corresponding segments within the samples. At the stage of assigning alleles to founders, gscramble provides a variety of ways to permute alleles among the collection of individuals from which the founders are drawn. By using permutation, variability in the data is created, but, since it is done without sampling with replacement, it does not induce RISPI.

These permutations are implemented with the genotypes provided by the user. gscramble accepts genetic data as a matrix with N rows (one for each individual) and 2L columns (two alleles for each locus). If the data have been phased, then the phased alleles from the first haplotype should occupy the first entry for each locus within an individual, while alleles phased together on the second haplotype should occupy the second entry for each locus. There can be several populations represented in the data set, but each individual must belong to exactly one population.

Permutation of alleles proceeds within populations, such that the frequencies of alleles from each population remain the same as those observed in the samples themselves. Following permutation, there remain individuals in each population, but the alleles that they carry are different after permutation. These individuals are assigned, in order, to be the founders from each population in the GSP. Subsequently, after genetic material is segregated to the samples, the alleles found on each genomic segment in those permuted founders are then propagated to the samples. It is worth noting that this approach allows for simulation with large genomic data sets, with hundreds of thousands to millions of markers, since segregation through the pedigree is done by segments, and not on a locus‐by‐locus basis. The only operations involving all the alleles in all the individuals are the permutation steps and indexing into the segments, which may be done efficiently with matrices in R.

**FIGURE 3:**
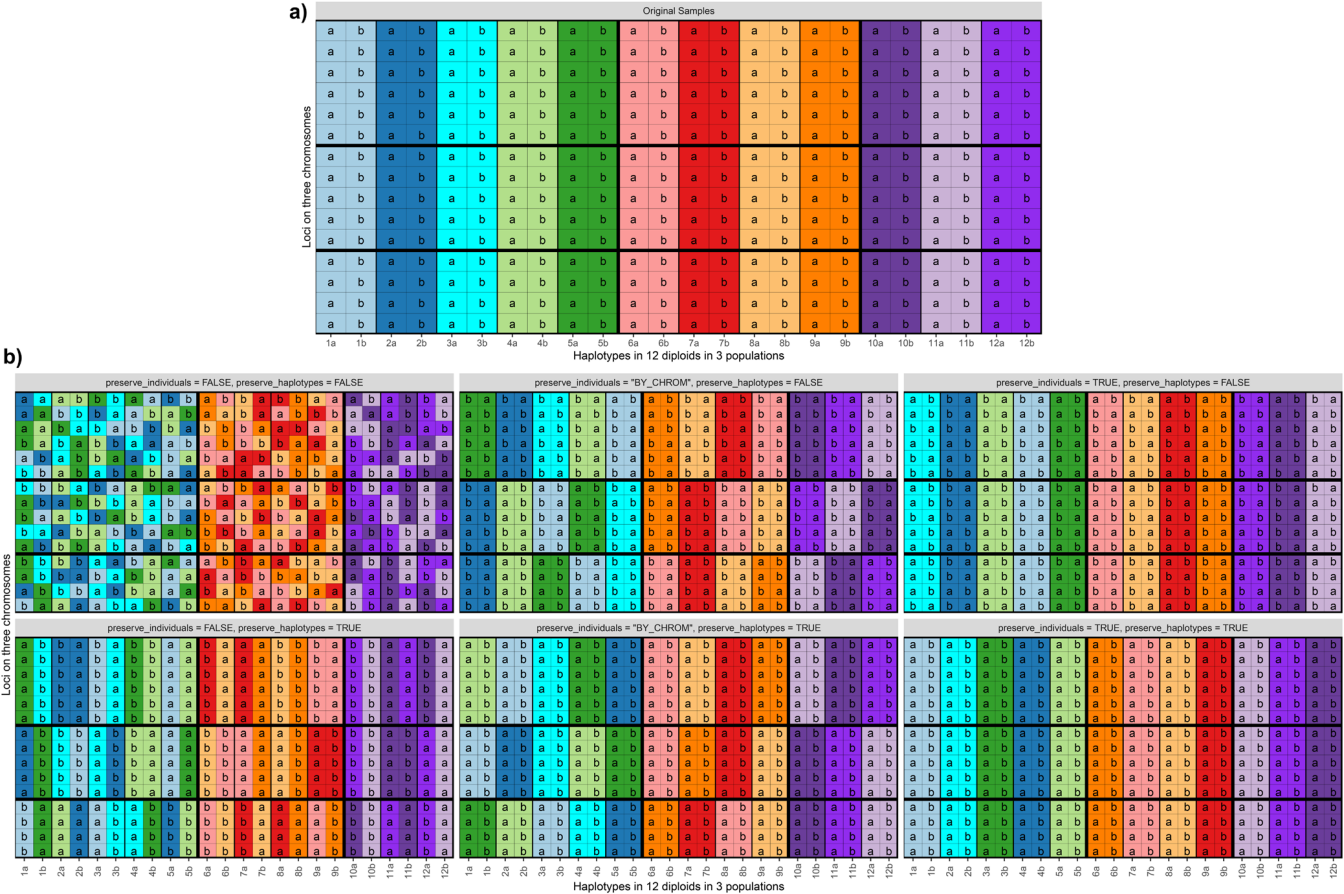
An illustration of how gscramble permutes data under different options. **(a)** A picture representing an original data set of *N* = 12 diploids sampled from three populations and typed at *L* = 15markers. Different colours represent different sampled individuals. Individuals 1–5 are shades of blue and green, indicating that they are from a single population. Individuals 6–9 are from another population, (shades of red and orange), while 10–12 are from a third population and are coloured shades of violet. Thick vertical black lines separate the different populations. Markers are shown in rows. There are 6 markers on the first chromosome, 5 on the second, and 4 on the third. Each chromosome is delimited by a thick, horizontal black line. The “a” and the “b” in the coloured cells denote the first and second gene copies within an individual at each marker. It is worth noting that this representation shows a 2 *L × N* matrix, which is the transpose of the *N ×* 2 *L* matrix used to supply genetic marker data to gscramble. **(b)** Examples of how the preserve individuals and preserve haplotypes options of the segments2markers() function affect the permutation of the genetic data of the original data set. Panel headers show the option values while the colours and “a” and “b” values in the coloured cells show the original position of each gene copy in the original sample. Note that permutation is always done within the populations. When preserve individuals = TRUE, then just the individuals in their entirety are permuted. When preserve individuals = “BY CHROM”, each pair of a single chromosome within an individual remains together when permuted. When preserve individuals = FALSE, gene copies are freely permuted between individuals. The option preserve haplotypes = TRUE causes each haplotype on a chromosome to be preserved while permuting. This option should only be used when the original data set is phased.

Permutation of alleles is handled within the gscramble function segments2markers() which takes as input the segments segregated down a GSP, a matrix of sampled genotypes, and a specification of the group that each sampled individual belongs to. The permutation performed when running segments2markers() is controlled by two main options. The first, preserve_individuals, can take three different values. If preserve_individuals = TRUE, then entire individuals are the unit that gets permuted within the data set, such that all the alleles within an individual remain together. If preserve_individuals = “BY_CHROM”, then the alleles within an individual found on a homologous pair of chromosomes remain together, but the different chromosome pairs of an individual are independently permuted into new positions in the data set. This form of permutation maintains the linkage disequilibrium (LD) between markers on the same chromosome, which may be desirable in some contexts. If preserve_individuals = FALSE then each gene copy within an individual is permuted separately, providing the maximal scrambling of the data. The second option, preserve_haplotypes, can take two different values, FALSE (the default) and TRUE. This option, which keeps alleles together on the same haplotype, should be set to TRUE only if the original genotypes have been phased into separate haplotypes. Figure 3 depicts, graphically, the effect of using different settings of preserve_individuals and preserve_haplotypes, together.

### 2.4 Examples

We illustrate the use of gscramble in three example cases. The first is a simple simulation to show that, unlike sampling with replacement, the approach of gscramble does not produce RISPI. The next two are examples using empirical data sets. The first of these involves simulating a small number (< 70) of markers that are fixed, or nearly fixed, for alternate alleles between two species of trout: steelhead trout ( *Oncorhynchus mykiss* ) and cutthroat trout ( *O. clarkii* ). The goal of the simulation study is to determine how accurately individuals of different hybrid categories between the species can be identified, and whether the use of linked markers deflates the estimates of uncertainty to an unacceptable degree. The second example involves many more markers (≈14,000) and investigates the accuracy for identifying admixed individuals of different hybrid classes between closely related groups of invasive wild pigs in Missouri.

#### 2.4.1 No RISPI When Using gscramble

For this example, we repeat the simulation described in section 2.1.1; however, after simulating individuals in the two samples drawn from the same population, we create data sets of admixed individuals using gscramble, rather than sampling gene copies with replacement. As before, individuals were simulated with fractions of ancestry from sample A in $\left\{0,\frac{1}{8},\frac{1}{4},\frac{3}{8},\frac{1}{2},\frac{5}{8},\frac{3}{4},\frac{7}{8},1\right\}$, but these were simulated from pedigrees using gscramble. We limited this simulation to one of the scenarios that showed extreme RISPI when sampling gene copies with replacement: L=1,000 markers with sizes of samples A and B being N=50. This simulation was repeated 240 times, and the results of each replicate were analysed with ADMIXTURE to estimate the ancestry fractions of each simulated individual.

#### 2.4.2 Steelhead/Cutthroat Trout Hybrids in California

We used the archived data from Rizza et al. (2023), drawing upon their 65 markers that are fixed or are nearly fixed for alternate alleles between steelhead trout and cutthroat trout. They designated 634 fish as pure cutthroat trout and 213 as pure steelhead. We used the genotypes of these individuals as input to gscramble.

Rizza et al. mapped the 65 markers to the Omyk_v1.0 genome assembly (RefSeq Accession: GCF_002163495.1), so we used the linkage map described in (Pearse et al. 2019) that was prepared with ≈57,000 markers described in Palti et al. (2015) mapped to Omyk_v1.0. We mapped these ≈57,000 markers to Omyk_v1.0, and then resolved inconsistencies to produce a map of 32,923 markers throughout the genome and rendered it to plink format for use with gscramble.

In the original paper, Rizza et al. (2023) used 30 of the 65 markers that occur on different chromosome arms to identify 15 $F_{1}$s, 1 $F_{2}$, 6 cutthroat backcrosses (*BC*_1_–CCTs) and 6 second‐generation cutthroat backcrosses (*BC*_2_–CCTs), and 1 fish of an unknown hybrid category that may have been the product of a ${\mathrm{BC}}_{2}$ crossed with a ${\mathrm{BC}}_{1}$. In our simulations, we sought to simulate a similar collection of hybrid individuals, so, for each replicate, we used gscramble to simulate 16 *F*_1_s, 4 *F*_2_s, 6 *BC*_1_s, 4 *BC*_2_s, 4 *BC*_3_s, 4 individuals that were the products of a ${\mathrm{BC}}_{1}$ mating with a *BC*_2_, and finally, four individuals that were simulated pure cutthroat. Individuals not used as founders in gscramble were retained as pure cutthroat and pure steelhead. The goal of the simulation was to assess how well these different hybrid categories can be distinguished from one another when using either all 65 markers, or just the 30 markers occurring on different chromosome arms used by Rizza et al. (2023).

We performed 500 replicate simulations with gscramble using either 30 or 65 markers. Each simulated data set was then analysed using newhybrids (Anderson and Thompson 2002). Individuals not used as founders were given the ‘s’ and ‘z’ flags, indicating that they were considered training samples. The remaining 46 individuals were treated as possible hybrids and their genetic data used to calculate the posterior probability that they belonged to one of 10 hybrid categories as shown in Table 1, using 25,000 MCMC sweeps and discarding the first 5000 as burn‐in.

**TABLE 1:** The 10 hybrid categories considered for inference with newhybrids and the expected frequency of their genotypes having 2 ($f\left(C,C\right)$), 1 ($f\left(C,S\right)$) or 0 ($f\left(S,S\right)$) copies of ancestry from cutthroat trout.

Category	$f\left(C,C\right)$	$f\left(C,S\right)$	$f\left(S,S\right)$
CCT	1	0	0
SH	0	0	1
$F_{1}$	0	1	0
$F_{2}$	0.25	0.5	0.25
${\mathrm{BC}}_{1}$–CCT	0.5	0.5	0
${\mathrm{BC}}_{1}$–SH	0	0.5	0.5
${\mathrm{BC}}_{2}$–CCT	0.75	0.25	0
${\mathrm{BC}}_{2}$–SH	0	0.25	0.75
${\mathrm{BC}}_{1}$–CCT × ${\mathrm{BC}}_{2}$–CCT	0.5625	0.375	0.0625
${\mathrm{BC}}_{1}$–SH × ${\mathrm{BC}}_{2}$–SH	0.0625	0.375	0.5625

#### 2.4.3 Invasive Wild Pigs in Missouri

Genetic samples were collected from 160 invasive wild pigs in southeastern Missouri that were lethally removed through population control efforts conducted as a component of the Missouri Feral Hog Elimination Partnership by US Department of Agriculture—Animal Plant Health Inspection Services—Wildlife Services, Missouri Department of Conservation, and other cooperative federal and state agencies. DNA was extracted from hair or tissue (collected from animals at the time of control) with commercially available magnetic bead recovery kits (MagMax DNA, Thermo Fisher Scientific). Genotyping was performed with GeneSeek's Genomic Profiler for Porcine HD which provides 62,128 biallelic SNP loci that are mapped across the 18 autosomal chromosomes. We implemented standard genotype quality control measures; specifically, we pruned loci with call rates <95% and minor allele frequencies <5%. We implemented LD pruning using a window size of 50 loci and step size of 5 loci to remove markers above a linkage threshold of $R^{2}>0.5$.

The 160 invasive wild pig samples represented 120 reference individuals from the core regions of 3 populations (40 individuals each informed by previous analyses) and 40 individuals from contact regions between these populations. We used ADMIXTURE version 1.3.0 (Alexander, Novembre, and Lange 2009) to characterise the genetic structure of the 160 samples (for K=1…6). Using the 120 reference individuals identified to represent population cores, we sought to contextualise the admixture patterns of the 40 samples from the contact regions using simulated pedigrees from gscramble. To address this objective we simulated pedigrees to represent the likely classes of admixed individuals that would be present in the samples from the contact regions of the 3 reference populations. The types of admixed individuals we considered were $F_{1}$, $F_{2}$, ${\mathrm{BC}}_{1}$ and ${\mathrm{BC}}_{2}$ representing all two‐population combinations (Pop1 x Pop2, Pop1 x Pop3, Pop2 x Pop3, and reciprocally Pop2 x Pop1, Pop3 x Pop1, Pop3 x Pop2). We also simulated individuals from each of the founder populations. The first step of this workflow involved simulating admixed individuals for 2 founder populations using one of the preset gscramble GSPs provided by the function create_GSP() for producing each of the types of admixed individuals. Although we could have simulated all 4 types of admixed individuals from a single GSP (Figure 2), different numbers of simulated individuals are produced for each hybrid class. Therefore, for ease of simulating the exact number of desired individuals, we chose to simulate from a single GSP for each of the hybrid classes by setting the corresponding hybrid category (i.e., F1, F2, etc.) parameter in create_GSP() to TRUE with all others set to FALSE. We used the default settings of the plink_map2rec_rates() function to specify the recombination rates. The 120 simulated genotypes were written to PLINK .ped and .map files using gscramble2plink(). We used PLINK version 1.9 (Purcell et al. 2007) to convert the genotypes to binary files and used these as input into ADMIXTURE for which we specified K=3 to produce ancestry values. We iteratively performed this workflow to produce 1000 simulated genotypes and ancestry values for each of the 4 hybrid classes, as well as founder individuals, for each of the population combinations, for a total of 21,000 simulated individuals.

## 3 Results

### 3.1 Two Introductory Motivating Examples

#### 3.1.1 RISPI When Estimating Admixture Fractions

**FIGURE 4:**
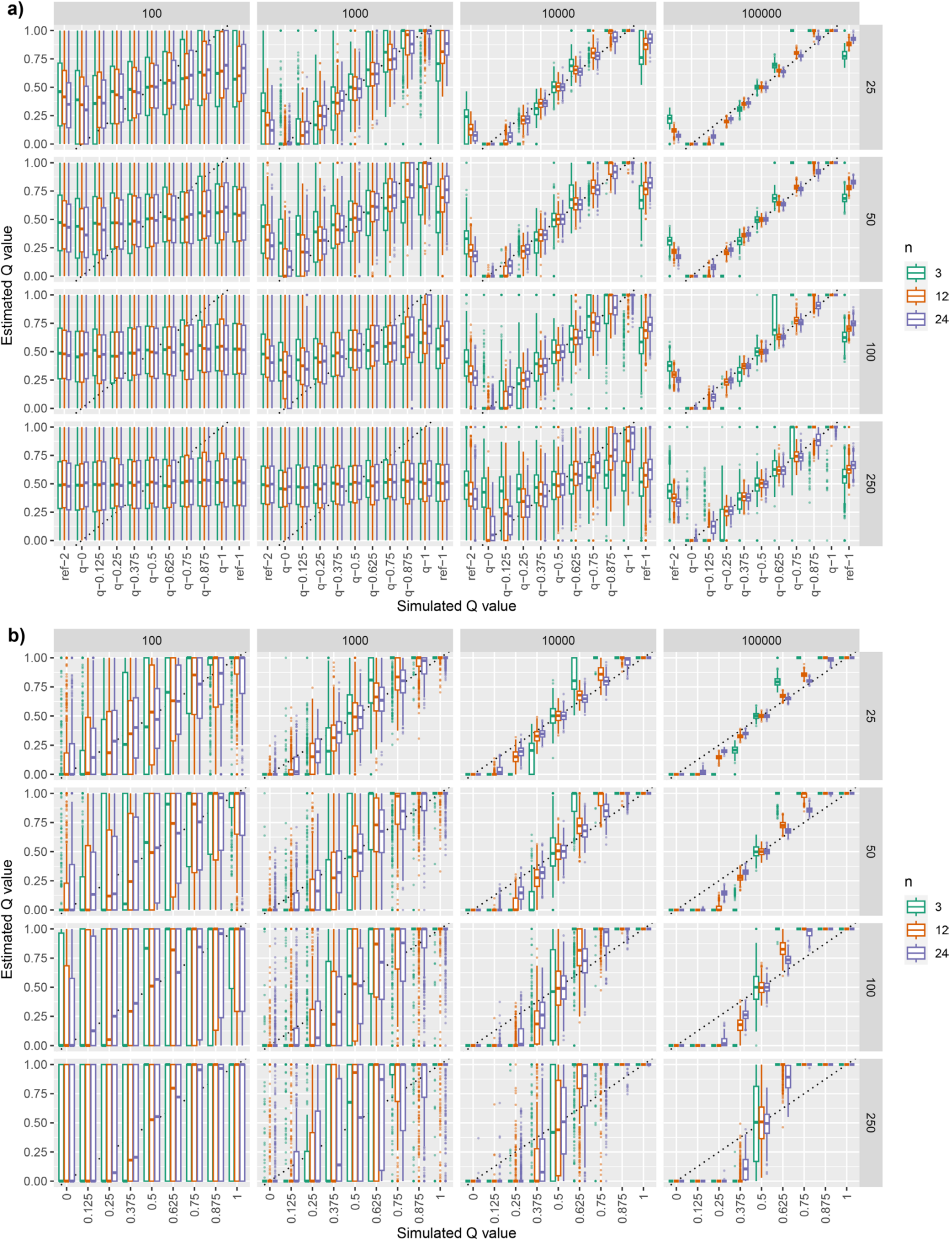
ADMIXTURE estimates of $q_{A}$ as described in *An introductory motivating example*. In these results, the apparent ability to estimate $q_{A}$ is a bias resulting from the use of sampling with replacement to simulate new, admixed genotypes to test in ADMIXTURE (e.g., these simulated samples suffer from RISPI). (a) Results from the supervised analysis in ADMIXTURE, where ‘ref‐2’ and ‘ref‐1’ show the *Q*‐values estimated by ADMIXTURE for the reference samples (even though they are treated by the program as being of known origin). (b) Results for the unsupervised analysis in ADMIXTURE. In both (a and b) the different columns represent different numbers of markers from 10 to 100,000, while different rows represent different original sample sizes, *N*, taken. Colours of the boxplots indicate how many new individuals, *n*, of each $q_{A}$ value were simulated during each replicate.

As expected, the perceived ability to estimate $q_{A}$ with ADMIXTURE was higher for larger values of L and for smaller values of N (Figure 4).

It is interesting to note that, for numbers of loci, L, that are 100 (or smaller) the effect is observed only when sample size, N, is as small as 25—and even then the effect is slight. However, when the number of markers increases to 100,000—values that are becoming commonplace with the availability of chip‐ or sequencing‐based approaches—it is apparent that the sampling‐with‐replacement approach induces an extreme bias. With 100,000 markers, even if sample sizes (N) are as large as 250 individuals, using naïve simulations improperly indicates that admixture fractions of individuals can be accurately estimated, even when there are no genetic differences between the populations from which samples A and B are drawn.

#### 3.1.2 Physical Linkage in Hybrid Individuals

**FIGURE 5:**
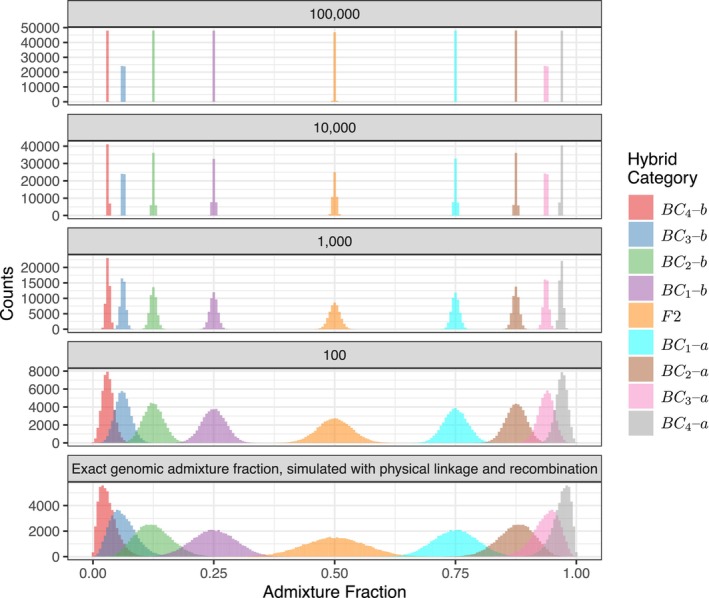
Admixture fractions of individuals simulated assuming no physical linkage (top four panels) and when physical linkage is simulated using gscramble assuming a genetic map with 18 chromosomes spanning 2.35 gigabases with recombination fractions like those found in pigs (Tortereau et al. 2012). In the top 4 panels the number of simulated loci appears in the panel header. Failing to simulate markers with physical linkage produces distributions of admixture fractions that are far more narrow than reality, giving the mistaken impression that admixture fractions of different hybrid categories do not overlap.

When hybrids were simulated in a way that accounts for the physical linkage of markers, the variance in admixture fractions was considerably higher than when markers were simulated under the assumption that none of them are physically linked (Figure 5).

If markers are assumed to be unlinked, then the variance around the Q estimates of different hybrid categories continually declines as more markers are added, from 100, to 100,000, which makes it appear that different hybrid categories become increasingly well‐resolved. For example, looking at the distributions for 100,000 unlinked markers one would think that it would be easy to distinguish between the ${\mathrm{BC}}_{1}$ through ${\mathrm{BC}}_{4}$ categories on the basis of the admixture fractions. This is, however, not the case. The bottom panel of Figure 5 shows the distribution of the true admixture fraction (calculated as the total length of genome from each of the two different species) of each individual when simulated with physical linkage using gscramble. When physical linkage is not ignored, it is quite clear that there is considerable overlap in the admixture fractions between the ${\mathrm{BC}}_{1}$ and ${\mathrm{BC}}_{2}$ categories, and that ${\mathrm{BC}}_{2}$, ${\mathrm{BC}}_{3}$, and ${\mathrm{BC}}_{4}$ all have admixture‐fraction distributions that overlap greatly. This shows that, in real life, when physical linkage cannot be ignored, it may be quite difficult to distinguish between different backcross categories on the basis of their admixture fractions.

### 3.2 Examples

#### 3.2.1 No RISPI When Using gscramble

**FIGURE 6:**
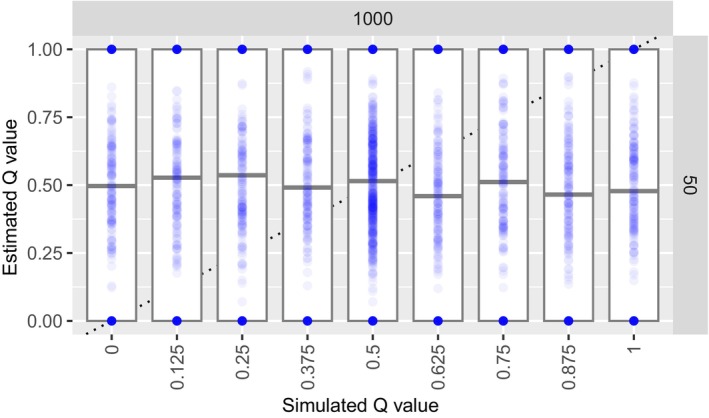
ADMIXTURE estimates of ancestry fractions, *Q*, for individuals simulated from samples from the same population, simulated using gscramble. There is considerable variation in the estimates, but the central tendency of them all is around 0.5, as would be expected in this case within which the different samples are drawn from the same population. Contrast this to the corresponding panel (*L* = 1, 000, *N* = 50) in Figure 4b, in which the RISPI is readily apparent.

As expected, there was no observed RISPI in the ADMIXTURE‐estimated ancestry proportions when simulating individuals using gscramble (Figure 6). On average, the estimated Q values were around 0.5, which is expected when the samples are drawn from the same population.

#### 3.2.2 Steelhead/Cutthroat Trout Hybrids in California

**FIGURE 7:**
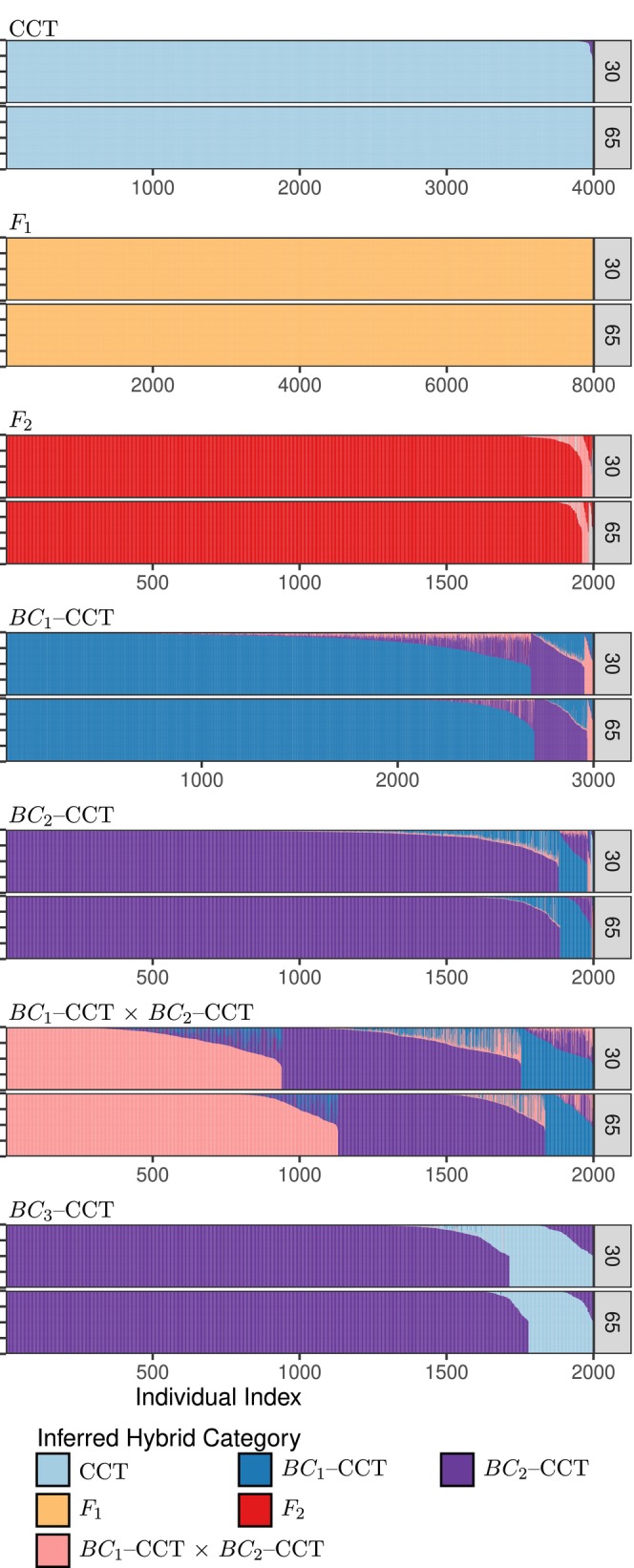
Posterior probabilities for simulated individuals of different trout hybrid categories. Each pair of plots shows the results for individuals of the true hybrid category listed atop the pair (see Table 1 for the meanings of the names). The top panel of each pair shows results for fish simulated with the 30 markers used by Rizza et al. (2023), and the bottom panel shows results for fish simulated with the original 30 markers, plus an additional 35 (65 total), some of which are linked on the same chromosome. Each individual appears as a thin vertical line, posterior probabilities of each inferred hybrid category are indicated by extent of different colours on those lines. Individuals are sorted according to maximum a posteriori hybrid category, and, within that by the maximum posterior probability.

The barplots in Figure 7 show the distribution of posterior probabilities of the different hybrid categories.

These are similar to STRUCTURE or ADMIXTURE barplots; however, rather than plotting the ancestry fraction, the different colours are showing the posterior probabilities of different hybrid categories. Each individual is represented by a thin vertical line, and the individuals have been arranged from left to right and the colours have been arranged from top to bottom throughout different sections of the plot to make it easy to see the patterns. The *maximum‐*a posteriori (MAP) hybrid category for any individual is represented by the colour that is on the bottom of the bar, so it is easy to see the shifts where the MAP hybrid category changes from one to another. It is apparent over all individuals simulated to be either CCT (cutthroat trout), $F_{1}$, $F_{2}$, ${\mathrm{BC}}_{1}$–CCT, or ${\mathrm{BC}}_{2}$–CCT, that the MAP estimate of their hybrid categories were correct for largely the same number of individuals whether 30 or 65 markers were used. By contrast, for the individuals simulated to be of the complex hybrid category ${\mathrm{BC}}_{1}$–CCT × ${\mathrm{BC}}_{2}$–CCT, the MAP estimate from 65 markers was correct more often than from 30 markers. However, it is also apparent that using 65 markers, some of which are physically linked, in the software *NewHybrids*—which assumes that all markers are unlinked—yields estimates of uncertainty that are inaccurate. Tellingly, when the estimates using 65 markers are incorrect (e.g., when ${\mathrm{BC}}_{1}$–CCT individuals have a maximum posterior of being in the ${\mathrm{BC}}_{2}$–CCT category, or when individuals that are truly of the ${\mathrm{BC}}_{1}$–CCT × ${\mathrm{BC}}_{2}$–CCT category have a maximum posterior of being from the ${\mathrm{BC}}_{2}$–CCT category), many of those incorrect posteriors are quite high (>0.90). In fact, using 65 markers yields more posteriors >0.90 for an *incorrect* hybrid category than does using the 30 unlinked markers. So, as expected, ignoring physical linkage (e.g., as implemented in newhybrids) can yield a poor estimate of the uncertainty around different inferences, and this analysis supports the choice of Rizza et al. (2023) to limit markers to those on different chromosome arms during their newhybrids analysis.

#### 3.2.3 Invasive Wild Pigs in Missouri

SNP filtering resulted in 14,233 loci that were used for subsequent analysis. Using the 160 empirical samples, ADMIXTURE results supported the identification of K=3 genetic clusters (Figure S1) that grouped individuals from each of the core populations into 3 clusters while the 40 individuals from the contact regions were highly admixed (Figure S2). The $F_{\mathrm{ST}}$ divergence from ADMIXTURE was: $F_{\mathrm{ST}}=0.24$ for Pop1‐Pop2, $F_{\mathrm{ST}}=0.14$ for Pop1‐Pop3 and $F_{\mathrm{ST}}=0.13$ for Pop2‐Pop3.

**FIGURE 8:**
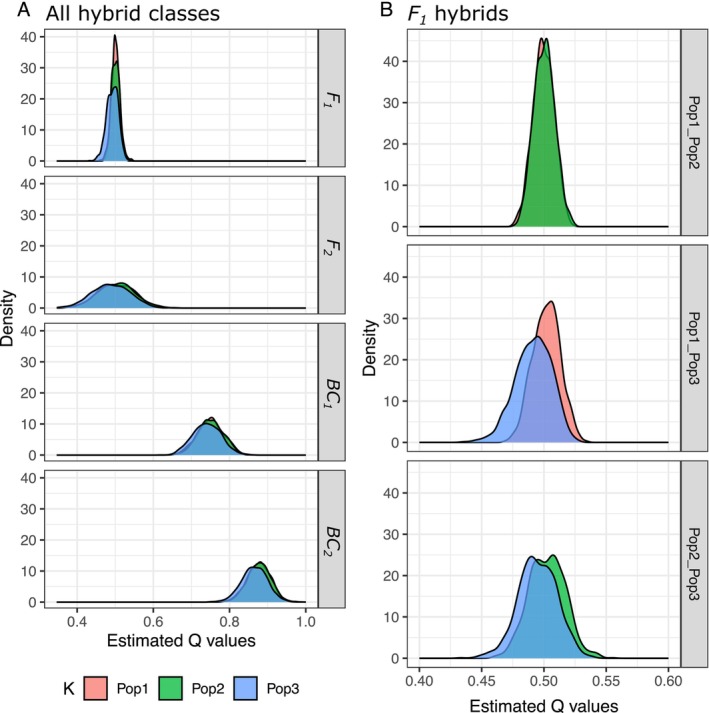
(A) The density of the estimated Q values from ADMIXTURE for the simulated runs of wild pigs are grouped across all population‐combinations for each hybrid class and coloured by the *K* genetic cluster (e.g., the Pop1 density plot for the *BC*_1_ plots the Pop1 genetic cluster for both the Pop1‐Pop2 and Pop1‐Pop3 combinations). The density plots across the *K* genetic clusters are mostly similar with the most variation among the genetic clusters evident in the *F*_1_ plot. (B) Plotting the density of the estimated Q values from ADMIXTURE for the *F*_1_ hybrids, separated by the population combinations, reveals the least variance in estimated Q values for Pop1‐Pop2 compared to Pop1‐ Pop3 and Pop2‐Pop3. Note that these population combinations group the reciprocal population combination in each plot (e.g., “Pop1 Pop2” has simulations from both Pop1‐Pop2 and Pop2‐Pop1).

The simulated hybrid classes of wild pig populations demonstrate how gscramble can be used to accurately produce the expected variation in the admixture fractions of different hybrid classes. $F_{1}$ and $F_{2}$ individuals had the expected range of admixture with mean Q=0.5, and $F_{2}$ individuals having more variation (Figure 8A). Backcrossed individuals have higher ancestry associated with the backcrossed population with mean Q approximately 0.75 (${\mathrm{BC}}_{1}$) and 0.87 (${\mathrm{BC}}_{2}$) (Figure 8A). Interestingly, the distribution of the Q values for $F_{1}$ hybrids varies more across the different genetic clusters than it does for the other hybrid categories. When the estimated Q values for the $F_{1}$ hybrids are separated by the population combinations, $F_{1}$ hybrids for Pop1‐Pop2 have the least variation around q=0.5 (standard deviation = 0.008), while Pop1‐Pop3 and Pop2‐Pop3 have higher variation (standard deviation = 0.011–0.016) (Figure 8B). Also, all Q distributions for $F_{1}$ hybrids, regardless of population combination, centre around 0.50 except for the two distributions of Pop3, which have a mean of Q=0.49 (Figure 8B). The differences among these distributions of Q values for the simulated data reflect the genetic differences observed in the empirical data, specifically, that genetic differentiation is highest between Pop1 and Pop2 and that Pop3 is the most admixed population with ancestry contributions from both Pop1 and Pop2. The results emphasise difficulties that may arise in estimating admixture fractions between groups that have experienced historical gene flow.

## 4 Discussion

Here, we present gscramble, an R package implementing a simulation approach for creating biologically informed individual genotypes of different hybrid classes. Two integral features of gscramble are: (1) alleles are sampled from populations without replacement and (2) alleles are segregated based on species‐specific recombination rates. Our results show that both of these features are especially important for accurately reflecting empirical data from large genetic marker sets (thousands of markers), which are common from both custom SNP‐chip genotyping and next generation sequencing methods. Sampling alleles without replacement, and thereby preserving the genetic differences (or lack thereof) of populations is essential for avoiding RISPI—an issue that is exacerbated as the number of genetic markers increases. Additionally, we demonstrate the importance of accounting for the physical linkage of markers when large marker sets are used, and that treating physically linked loci as independent erroneously reduces the variation in the admixture fractions of simulated individuals. In connection with this, we also stress that simply pruning markers to eliminate or reduce linkage disequilibrium (LD) will not solve the problem posed by physical linkage. Genomic material is inherited in chunks that are typically orders of magnitude longer than the typical range over which LD decays. Thus, pairs of markers that are on the same chromosome, are much more likely to be co‐inherited than markers that are not on the same chromosome, even if the two markers are far enough apart on the same chromosome that they are not in LD. That said, for some downstream analyses (after running gscramble), LD pruning can be advantageous; for example, ADMIXTURE assumes independent loci and will run faster on a smaller, pruned data set.

gscramble is suitable for analysis of numerous types of marker sets available from contemporary data generation techniques. As mentioned above, one key feature of gscramble is the integration of recombination rates, thereby accounting for physical linkage which is expected among high‐density markers sets. Furthermore, gscramble performs marker segregation with computational efficiency—for the four different GSPs used with the invasive wild pig data set, the mean run time of each simulation with 14,233 SNP loci was 2.3 s (range: 1.9–5.7 s). While gscramble is equally applicable to data sets with microhaplotypes (Baetscher et al. 2018) or microsatellites (Zhan et al. 2017), it is not built to use genotype likelihoods (i.e., from low‐coverage whole genome sequencing). Specifically, simulations conducted within gscramble involve the segregation of gametes within the specified pedigrees and, therefore, require called genotypes (though missing data is acceptable).

We envision gscramble to be relevant for a diverse range of potential applications. In our provided empirical examples with trout and wild pig data, we demonstrate the basics of creating hybrid classes. In much the same manner, gscramble has been used to evaluate the statistical power of hybrid classification used to differentiate wild boar hybrids from domestic pigs (Smyser et al. 2024). Specifically Smyser et al. (2024) used the segregation options in gscramble to preserve founder individuals' genotypes in contrast to a priori shuffling alleles within populations. This retains the subpopulation structure present within certain populations—thereby, better reflecting the actual admixture variation expected from wild boar and domestic pig hybrids. We think gscramble may also be useful for assessing relatedness estimates using pedigrees of varying complexity. Here, it is important to note that each time a gamete is segregated in a GSP, its complement is also segregated—an example of antithetical variate sampling in Monte Carlo methods (Hammersley and Handscomb 1964). Thus, in the example of relatedness, fixed specified founders should be repeatedly ‘mated’, with one offspring sampled per iteration to avoid sampling of anticorrelated genotypes.

As noted in the methods, the GSP used to conduct simulations with gscramble must not contain inbreeding loops, since the occurrence of such loops means genes would be sampled with replacement from the founders, potentially inducing RISPI and incorrectly inflating the perceived power for admixture fraction estimation. One might reasonably ask whether this limits the application of gscramble in small populations of conservation concern where inbreeding occurs; we submit that it does not. If genetic structure between two locales is driven by family structure (as it often may be in very small and inbred populations), that family structure will be reflected in samples taken from each locale—the samples within a locale may be close relatives of one another. If related individuals are used as founders in a GSP, then the offspring they produce will essentially be inbred because the founders are related even though the GSP contains no inbreeding loops. The inclusion of related founders in this way, then, also appropriately reflects the genetic drift that is causing allele frequency differences between the locales.

Here, we have demonstrated the consequences of failing to consider physical linkage among loci given the availability of a recombination map. However, in the absence of a recombination map, researchers with a basic understanding of the genome size and the number of chromosomes in the study organism could still benefit from using gscramble to simulate genetic data under an assumed, approximate linkage map. To do so, users of gscramble could randomly distribute markers across, and within, the known number of chromosomes to produce a conceptual marker map (*.map within the PLINK file structure), and assume basic rates of recombination (e.g., 1 cM per 1 Mb). The resulting simulations from gscramble would produce admixture fractions that capture the expected variation from known linkage patterns, in contrast to the reduced variation produced from assuming independence (Figure 5). Characterising uncertainty in this way would provide caution for researchers to not overinterpret conclusions drawn in the absence of a recombination map.

## Author Contributions

The gscramble simulation approach grew from discussions between Timothy J. Smyser and Eric C. Anderson. Eric C. Anderson, Rachael M. Giglio, Matthew G. DeSaix, and Timothy J. Smyser wrote the R package, ‘gscramble.’ Simulations of bias due to RISPI and of variance in admixture fractions due to physical linkage were conducted by Eric C. Anderson. The empirical analyses using ‘gscramble’ were conducted by Matthew G. DeSaix, Rachael M. Giglio, Timothy J. Smyser, and Eric C. Anderson with previously published data. All authors contributed to writing and editing the manuscript.

## Conflicts of Interest

The authors declare no conflicts of interest.

## Supporting information


Data S2

## Data Availability

A stable version of the ‘gscramble’ R package is available on CRAN: https://CRAN.R‐project.org/package=gscramble. The development version and entire revision history of gscramble is available on GitHub: https://github.com/eriqande/gscramble, and all package documentation is available online in HTML format: https://eriqande.github.io/gscramble. Data and code for the simulation analyses and steelhead‐cutthroat analyses are available from https://github.com/eriqande/gscramble‐paper‐sims‐and‐analyses and are archived at https://doi.org/10.5281/zenodo.14270660. Data and code for the pig analyses are available from https://github.com/mgdesaix/gscramble‐paper‐pigs and are archived at https://doi.org/10.5281/zenodo.14271038. *Benefit‐Sharing Statement*: Benefits from this research accrue from the sharing of our data and results on public databases and the distribution of a novel and useful R package as described above.
