# Supplementary material for: gscramble: Simulation of Admixed Individuals Without Reuse of Genetic Material
Source: Mol Ecol Resour. 2025 Jan 12;25(4):e14069. doi: 10.1111/1755-0998.14069 (PMC11969638; doi:10.1111/1755-0998.14069)

MOLECULAR ECOLOGY

Supplemental Information for:

GSCRAMBLE: Simulation of admixed individuals without reuse of genetic material

Eric C. Anderson, Rachael M. Giglio, Matthew G. DeSaix, Timothy J. Smyser

Table of Contents:

**Figure S1: Admixture cross-validation**	Page 1
**Figure S2: Admixture Q plot**	Page 2

MOLECULAR ECOLOGY

Figure S1. Cross-validation errors for the clustering analysis of empirical invasive wild pig data with ADMIXTURE were produced using ADMIXTURE's default 5-fold cross-validation procedure (--cv=5) for values of K=1-5. At K=3, the cross-validation error began to flatten around an error of 0.5.

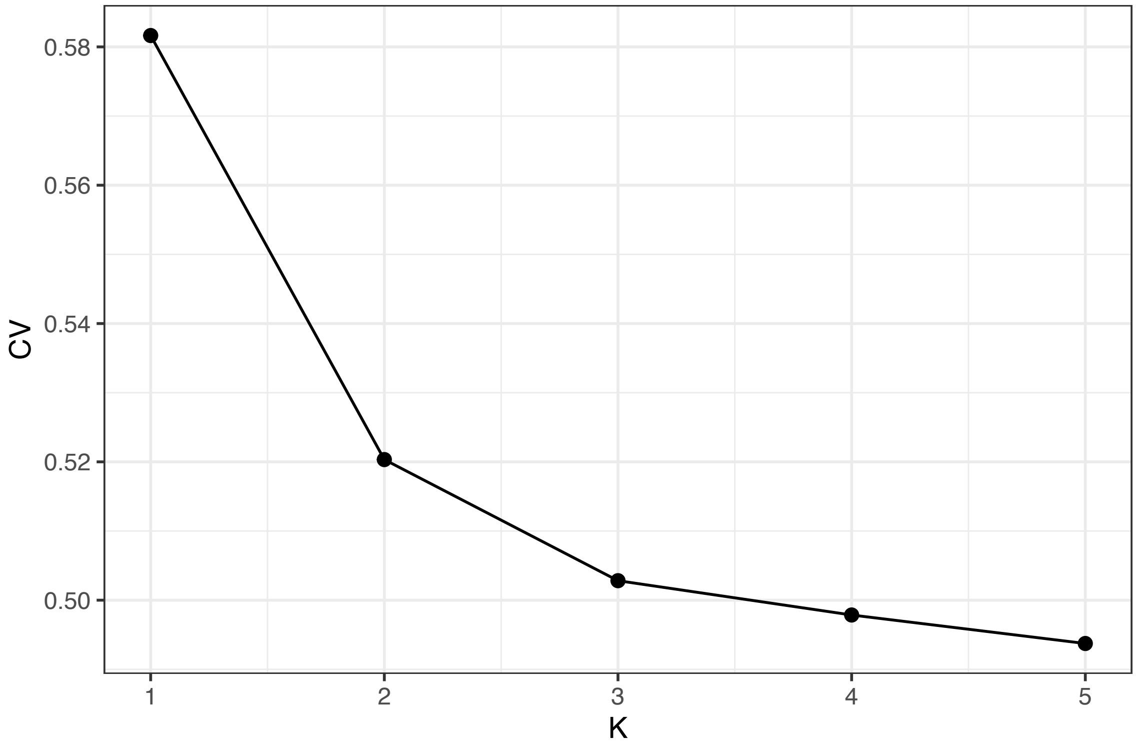

MOLECULAR ECOLOGY

Figure S2. ADMIXTURE Q plot for K=3 of the empirical invasive wild pig data (n = 160). Individuals from Pop1 clustered most cohesively together, while Pop2 and Pop3 were increasingly admixed, but still predominantly represented by a single ancestral group (K2 and K3, respectively). As expected, individuals from the "Contact" region, were highly admixed between all three clusters.

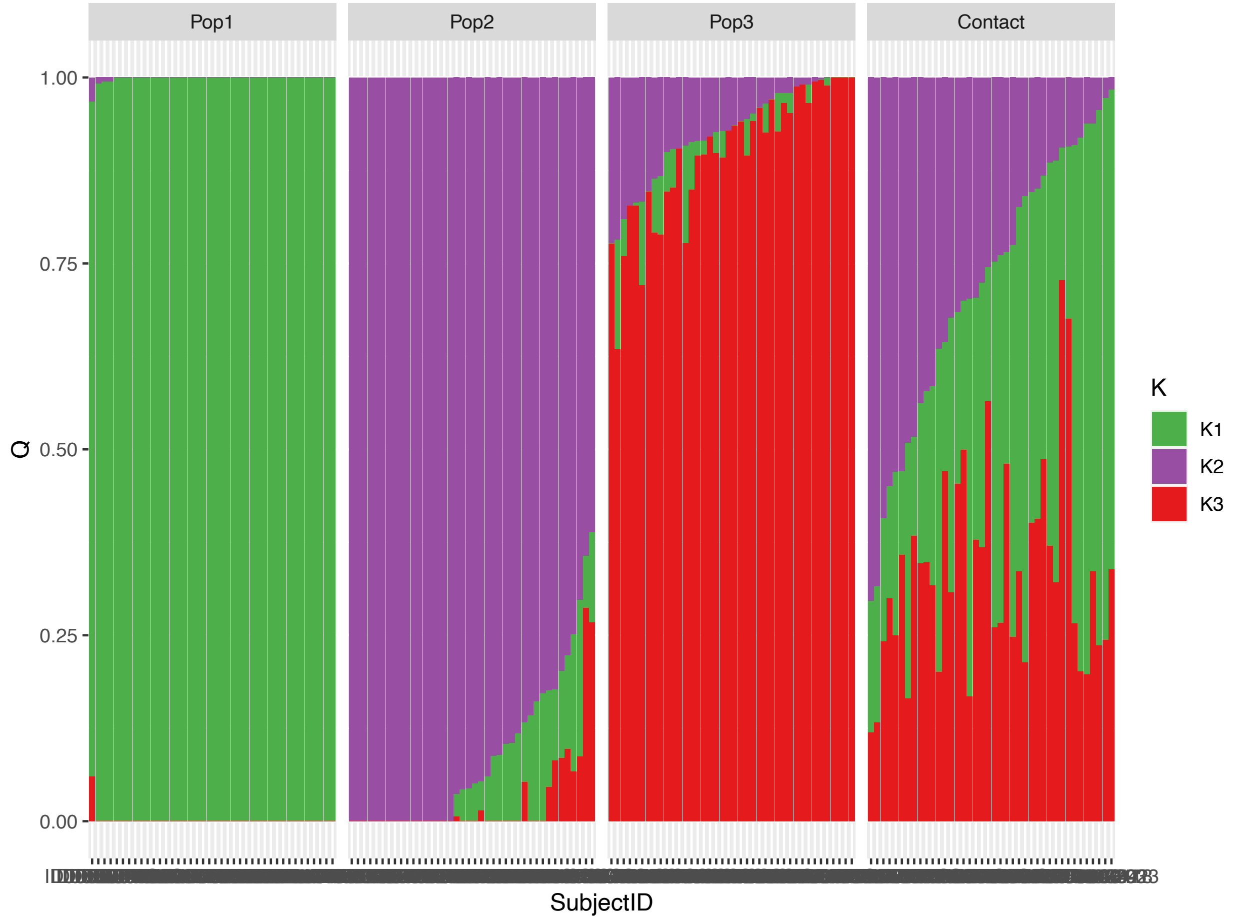

Supplement: Supplementary file 1 — Data S2 [file MEN-25-e14069-s001.pdf]
